# Community-Engaged Modeling of Geographic and Demographic Patterns of Multiple Public Health Risk Factors

**DOI:** 10.3390/ijerph14070730

**Published:** 2017-07-06

**Authors:** Komal Basra, M. Patricia Fabian, Raymond R. Holberger, Robert French, Jonathan I. Levy

**Affiliations:** 1 Department of Environmental Health, Boston University School of Public Health, Boston, MA 02118, USA; pfabian@bu.edu (M.P.F.); jonlevy@bu.edu (J.I.L.); 2 Office of Environmental Stewardship, City of New Bedford, New Bedford, MA 02740, USA; Raymond.Holberger@newbedford-ma.gov; 3 NorthStar Learning Centers, New Bedford, MA 02740, USA; rfrench@northstarlc.org

**Keywords:** GIS, spatial microsimulation, community partnerships, diabetes, exercise

## Abstract

Many health risk factors are intervention targets within communities, but information regarding high-risk subpopulations is rarely available at a geographic resolution that is relevant for community-scale interventions. Researchers and community partners in New Bedford, Massachusetts (USA) collaboratively identified high-priority behaviors and health outcomes of interest available in the Behavioral Risk Factor Surveillance System (BRFSS). We developed multivariable regression models from the BRFSS explaining variability in exercise, fruit and vegetable consumption, body mass index, and diabetes prevalence as a function of demographic and behavioral characteristics, and linked these models with population microdata developed using spatial microsimulation to characterize high-risk populations and locations. Individuals with lower income and educational attainment had lower rates of multiple health-promoting behaviors (e.g., fruit and vegetable consumption and exercise) and higher rates of self-reported diabetes. Our models in combination with the simulated population microdata identified census tracts with an elevated percentage of high-risk subpopulations, information community partners can use to prioritize funding and intervention programs. Multi-stressor modeling using data from public databases and microsimulation methods for characterizing high-resolution spatial patterns of population attributes, coupled with strong community partner engagement, can provide significant insight for intervention. Our methodology is transferrable to other communities.

## 1. Introduction

There is a need to characterize patterns of risk factors within communities at a resolution that is meaningful to community-level public health efforts. Geographic information systems (GIS) have become a popular and powerful tool in community health [1], especially when publicly available databases have been leveraged. Public health and population health studies have been undertaken to address this need, including through the development of various spatial microsimulation methodologies in the US, UK, New Zealand, and Australia on topics such as poverty, obesity, smoking, and mental health [2,3,4,5,6]. In this context, we define spatial microsimulation as the use of simulation methods to generate individual-level data at higher geographic resolution than available in publicly available administrative datasets. While these studies provided important insights at a high geographic resolution, these spatial microsimulation methods have not typically been developed or applied to respond directly to community members’ or community-serving organizations’ needs.

Developing meaningful models within a community context requires a shift from conducting research on communities to working collaboratively on topics of mutual interest [7]. Zip code level community-engaged plans were developed in Florida to address infant mortality [8] and in Michigan [9] to tackle diabetes prevalence. While these studies provided important insights relevant to public health policy and practice, the low geographic resolution makes the data less nuanced than studies utilizing spatial microsimulation techniques. When attempting to determine optimal intervention strategies or plans within a city, data with greater geographic and demographic resolution are required, but studies have not previously generated this information following a process that would most meaningfully inform community decision-making.

In this study, we focused on New Bedford, Massachusetts (MA), USA, a low-income city with multiple environmental and public health challenges. Given limited resources and a diverse population, within-city comparisons are crucial to local government and community-based organizational needs assessments and planning. We leveraged our previously developed spatial microsimulation methodology to predict high-resolution patterns of multiple risk factors by: (1) constructing a simulated population with multivariable demographic attributes at high spatial resolution (synthetic microdata), and (2) building regression models using public databases to predict risk factors of concern to community partners as a function of available demographic information [10,11]. To our knowledge, this is the first application that has directly connected spatial microsimulation methods with high-priority needs articulated by the community in a fully collaborative context.

## 2. Materials and Methods

### 2.1. Study Area

New Bedford is a city located in southeastern MA, with a population of approximately 95,000 according to the 2010 US Census. 17% are Hispanic/Latino (10% statewide), 43% live in owner-occupied housing (62% statewide), 37% speak a language other than English at home (22% statewide), 30% are less than high school educated (10% statewide), 13% are living with a disability (8% statewide), and 24% live in poverty (12% statewide) [12,13]. 70% of the population in New Bedford lives in census block groups meeting one of the MA environmental justice criteria (i.e., low median household income, high percentage of minority, or limited English proficiency) [14].

### 2.2. Methodology

#### 2.2.1. Construct New Bedford Synthetic Microdata

Our spatial microsimulation methodology for constructing the synthetic microdata has been published previously [10]. Briefly, the synthetic microdata were developed by combining individual-level microdata with coarse geographic resolution (Public Use Microdata Area) with census tract-level constraints from the American Community Survey (ACS), applying probabilistic reweighting using simulated annealing to optimize the assignment of individuals to census tracts. New Bedford includes 31 census tracts, with a mean population of 3063 (range: 1448–5591). The census tract-level constraints included eight individual-level constraints (ancestry, sex, age, educational attainment, sex by age, sex by educational attainment, age by educational attainment, and sex by age by educational attainment) and five household-level constraints (head of household age, household income, rent/own, rent/own by head of household age, household income by head of household age). This resulted in a database of 94,944 individuals representing New Bedford residents, populated with individual sociodemographic and housing characteristics at a census tract resolution.

#### 2.2.2. Prioritize Health Outcomes and Risk Factors

Community partner organizations on the team included a non-profit organization, NorthStar Learning Centers, with the mission to assist and empower youth to overcome adversity resulting from poverty, educational disadvantage, and other stressors so they may transform their lives and communities, and a local municipal governmental agency, the Office of Environmental Stewardship, which directs the City’s environmental compliance and planning efforts. The university and community partners first began working together in 2010, as collaborators on an EPA STAR grant on the topic of incorporating nonchemical stressors into cumulative risk assessment. The collaboration was initiated given a mutual interest in attention deficit hyperactivity disorder and its impact on schools and children. Throughout that grant, we held monthly team meetings and community partners provided regular input to study design and implementation, including extensive survey data collection. At the end of the funding period, we formally reflected on our model for community engagement [15] and concluded that while we had developed a strong partnership and expanded the scope of topics of interest to the community partners, there was a need to conduct action-oriented work led by community partner interests subsequent to the end of the grant.

The research team discussed the possibility of leveraging the synthetic microdata to address high-priority needs defined by the community partners. Subsequent to some brainstorming conversations, we determined that the most relevant database given the domains of community interest would be the Behavioral Risk Factor Surveillance System (BRFSS), a national telephone survey of adults ages 18 and older used to collect information yearly on a wide range of health risk behaviors and health conditions [16]. We then developed a formal collaborative process to select outcomes from the BRFSS. The university partners generated information on all relevant questions from the BRFSS, and we held multiple meetings to determine the subset of questions of greatest interest. Criteria for selecting outcomes included: relevance and importance to community partners, modifiable behaviors or interventions, and potential for funding to implement interventions. The three behaviors/outcomes selected included exercise, fruit and vegetable consumption, and diabetes. We also modeled self-reported body mass index (BMI), given a strong association with diabetes and other outcomes.

#### 2.2.3. Literature Search for Candidate Predictors

We conducted a literature search in September 2015 to identify predictors of exercise, fruit and vegetable consumption, BMI, and diabetes. We conducted searches in Google Scholar, PubMed, and Web of Science, using search terms such as the four health outcomes/behaviors and ‘predictor’ or ‘risk factor’ to identify articles of interest. A list of candidate variables was compiled from these articles, and we selected the subset that were available both in the BRFSS and the New Bedford synthetic microdata.

#### 2.2.4. Regression Modeling

All regression models were built using data from the 2005–2010 MA BRFSS, a database of individual observations that includes variables on health risk behaviors, health conditions, and demographics. We built regression models from Bristol County data (*n* = 15,814), which is inclusive of New Bedford (the largest city in Bristol County). We modeled the three primary behaviors/outcomes of interest by building multivariable logistic regression models from questions in the BRFSS for exercise (did you do any exercise in the past 30 days?), fruit and vegetable consumption (do you eat five more servings of fruit or vegetable a day?), and diabetes prevalence (have you ever been told by a doctor that you have diabetes?). We also built a multivariable linear regression of BMI as a function of demographic and behavioral variables listed in Table S1. All questions and covariates were based on the structure within the BRFSS, with categories collapsed for a subset of questions (e.g., income) with sparse data in some categorical responses. Models were constructed by backward selection using SAS statistical software version 9.4 (SAS Institute Inc., Cary, NC, USA). The criterion for dropping a variable was a p-value of greater than 0.05. We tested the sensitivity of our findings by applying the same model to BRFSS data restricted to New Bedford only (*n* = 4399).

#### 2.2.5. Outcome Prediction

We predicted probabilities of any exercise in the past 30 days, of eating five or more servings of fruits or vegetables daily, of BMI categories (continuous BMI was predicted and then categorized), and of diabetes status for each individual in the synthetic microdata by applying the multivariable regression models (Section 2.2.4) to the synthetic population (Section 2.2.1). The synthetic population includes individual-level demographic variables (e.g., age, gender, race/ethnicity, education, income) as well as predicted variables for smoking and alcohol use, at census tract resolution [10]. The BRFSS collects information from individuals 18 and older, however our study synthetic population was restricted to include only individuals who could legally consume alcohol, thus age 21 and older.

To quantify uncertainty for each of the binary outcomes for which probabilities were assigned, a Monte Carlo simulation was run using @Risk version 7 (Palisade Corporation, Ithaca, NY, USA) (uncertainty was not quantified for BMI). Each model was simulated 1000 times, and individual coefficients of variation around each predicted binary outcome at the census tract level were calculated. To evaluate the internal consistency of our models, we compared our aggregated predictions to city-wide measures reported by the Massachusetts Department of Public Health (MA DPH) for BRFSS data from 2005 to 2010 for New Bedford.

#### 2.2.6. Outcome Presentation

Predicted individual exercise, fruit and vegetable consumption, and diabetes status were aggregated by census tract and mapped using ArcMap 10.1 (Esri, Redlands, CA, USA). Maps as well as data tables were refined with community partners to maximize applicability to community needs. For example, community partners provided feedback on map aesthetics and on questions for which maps would be more or less valuable than tabular presentations of key statistics. Refined versions of the maps along with supporting tables were then presented to other interested community stakeholders.

## 3. Results

### 3.1. Literature Search Results

We reviewed 45 publications from the literature to identify established predictors of exercise, fruit and vegetable consumption, BMI, and diabetes (publications are listed in the bibliography in the supplemental material). Candidate predictors are listed in supplemental Table S1 and indicate those variables found in the literature that were also available in the BRFSS and the New Bedford synthetic microdata. Predictors selected to build the regression models were sex, age, race/ethnicity, income, education, employment, smoking status, alcohol consumption, exercise, diet, and BMI.

### 3.2. Regression Model Results

Our regression model for probability of exercising in the past 30 days, based on data from Bristol County (base population *n* = 15,814), showed that being female, Hispanic, lower income, less educated, a current smoker, or not having at least one alcoholic drink in the past 30 days were associated with being less likely to have partaken in any exercise in the past 30 days (Table 1). Being male, less educated, a current smoker, or not exercising in the past 30 days were associated with being less likely to eat five or more servings of fruits or vegetables daily (Table 1). Being male, lower income, less educated, not having at least one alcoholic drink the past 30 days, not exercising in the past 30 days, or not consuming five or more serving of fruits or vegetables daily were associated with a higher BMI (Table 1). Lastly, adults who were male, older, low income, a former smoker, did not have at least one alcoholic drink in the past 30 days, did not exercise during the past 30 days, or were obese were more likely to have been told by a health professional that they had diabetes (Table 1). Findings were similar when regression models were built from BRFSS data restricted to observations from New Bedford (base population *n* = 4399) (Table S2).

The multivariable regression models reveal which groups of adults are at high risk for the multiple risk factors explored. For example, lower income individuals were less likely to exercise and were more likely to have a higher BMI and have diabetes. Less educated individuals were less likely to exercise or eat five or more servings of fruits or vegetables, and were more likely to have a higher BMI. Patterns were more complex for some subpopulations. While men were less likely to consume five or more servings of fruits and vegetables, women were less likely to exercise. Current smokers were less likely to exercise and consume five or more servings of fruits and vegetables, but had a lower BMI and were less likely to have diabetes. In general, older and obese low-income men were the highest-risk population for diabetes. Importantly, these predictors do not necessarily reflect causal pathways, but rather represent clusters of behaviors and demographic characteristics that are often found together—in other words, smoking is not protective against diabetes, but diabetics are less likely to be current smokers given medical recommendations and potential adaptive behaviors.

### 3.3. Identification of Census Tracts with Populations at High Risk for Modeled Outcomes

**Figure 1:**
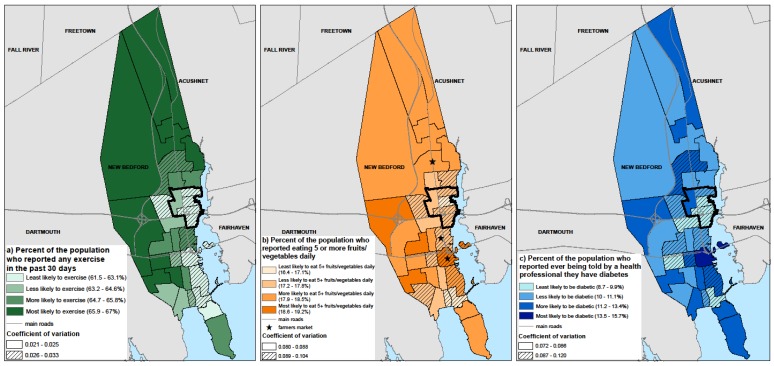
(**a**–**c**) Predicted exercise, fruit and vegetable consumption and diabetes prevalence by census tract in New Bedford, MA estimated using synthetic population microdata. Black outlined census tracts were identified as high priority by community partners. Coefficients of variation around each predicted outcome at the census tract level were calculated by running a Monte Carlo simulation of each model 1000 times to quantify uncertainty. Data sources: exercise, fruit/vegetable and diabetes proportions modeled from MA BRFSS 2005–2010 and applied to synthetic population microdata [10].

The regression models were applied to the synthetic microdata, and the resulting patterns by census tract were mapped for exercise, fruit and vegetable consumption, and diabetes prevalence (Figure 1a–c). We examined overall spatial patterns with specific emphasis on four census tracts of a priori interest to community partners. These census tracts were entirely compromised of census tract block groups that met one or more of the MA environmental justice criteria (i.e., low median household income, high percentage of minority, or limited English proficiency [14]), and they were of particular interest for ongoing Brownfields Area Wide Planning efforts and an upcoming brownfields grant submission by the community partners. Exercise prevalence was lowest in eastern central and southeast New Bedford (Figure 1a). Similar patterns were seen for fruit and vegetable consumption; farmers markets were located in census tracts with relatively higher consumption rates (Figure 1b). Unlike exercise and fruit and vegetable consumption prevalence, census tracts with the highest diabetes prevalence were not concentrated within one area of New Bedford, consistent with the importance of age as a predictor (Figure 1c). Exercise and fruit and vegetable consumption rates for all four census tracts of interest to community partners are below the city average. Diabetes prevalence is generally lower than the city average, consistent with the younger and more female populations in those tracts.

### 3.4. Evaluation of Results

The proportion of people who exercise, consume five or more servings of fruits and vegetables, and have diabetes were computed for our synthetic population and compared to proportion data reported by the MA DPH [17], and in all three cases the 95% confidence intervals overlapped (Table 2). The corresponding values for the state of Massachusetts reinforce the greater risk of poor health in New Bedford than statewide. A higher percentage of the population in Massachusetts exercises and eats five or more servings of fruits and vegetables compared to the population in New Bedford, and diabetes prevalence is lower statewide.

## 4. Discussion

As expected, significant predictors in our models matched those found in the literature. For example, Boutelle et al. (2004) found that exercise was associated with ethnicity, income, and smoking [18]. Trudeau et al. (2003) found that differences existed between predictors of fruit and vegetable consumption by gender, and predictors included age, education, exercise, and smoking [19]. In the literature, employment [20] as well as income and education were found to be significant predictors of BMI [21]. Lastly, predictors of diabetes included demographics, BMI, diet, and lifestyle indicators [22]. In spite of the number of highly significant predictors, only a small fraction of variability in these behaviors and outcomes can be explained by basic sociodemographic predictors. For example, the majority of the exercise literature examines predictors such as family support, attitudes, and environmental factors [23,24], and a systematic review of the literature on fruit and vegetable consumption found that the majority of the literature examines predictors—including intentions, attitudes, perceived barriers, and autonomy [25]—which are more specific predictors not readily available in publicly available datasets. That said, our ability to determine sociodemographic predictors of key behaviors and outcomes allowed us to connect with a population dataset developed through spatial microsimulation, thereby yielding predictions of within-city variability not otherwise available.

In conversations between university and community partners, community partners indicated value in determining factors that show the needs of specific New Bedford census tracts in comparison to elsewhere in New Bedford, the rest of Massachusetts, or the nation. The results can be used to help target planning efforts on and within particular neighborhoods. Specifically, annual applications for brownfield grants to further the city’s environmental justice efforts are augmented by providing data-driven demographically and spatially resolved insights in their applications. Similarly, grant applications to expand the farmers market program are greatly informed by spatially resolved insight about high-risk subpopulations. The modeled data provide needed information to focus health promotion efforts at a geographically resolved level that was not previously or publicly available to government agencies, non-profits, or community groups. The novelty of this application is most markedly apparent in Figure 1, which conveys variability in need across New Bedford, along with the marked differences with the state as a whole (Table 2). Additionally, the regression models (Table 1) point toward specific demographic groups that could be targeted, either independent from or in conjunction with spatially-oriented interventions. For example, lower income individuals with lower educational attainment in New Bedford had lower rates of multiple health-promoting behaviors and higher rates of diabetes.

The spatial microsimulation and linked regression modeling methodology used in New Bedford can be applied to any community across the US, given that we relied on public databases available for any location, and could generalize to other countries given analogous census data and representative population surveys. While needs vary across communities, data-driven insights about geographic and demographic patterns of modifiable health behaviors within communities can be used to create or enhance current community programming and interventions, obtain funding for interventions, or to support policy and advocacy efforts.

While the analytic methods in our study are generalizable and have demonstrated utility in the public health literature, another key dimension of our project involved strong community partner engagement, which allowed us to design and implement statistical models that could directly inform community-scale public health programs. This type of engaged research involves a shift from research on communities (one-way) to bi-directional community engagement in which researchers share power and conduct studies with communities [7]. The benefits of bi-directional community engagement are clear: the researchers develop a better understanding of, communication with, and connection to the community, and the community benefits from the research, tools, and technical expertise, enhancing social capital as well as community empowerment [26]. While there are clear challenges—including initiating, maintaining, and developing a relationship with a community that includes diverse stakeholders, and balancing competing priorities and expectations [27]—community-engaged research ultimately leads to improved research quality, community relevant research, and impactful research that addresses health disparities [28]. The demonstrated tools and data can lead to more targeted programming, better allocation of resources, enhanced community decision-making, and overall improved health of communities.

One limitation of the study is the number of predictors available for the regression models, shown in Table S1. We were constrained to using only variables available in the synthetic population, which was derived from ACS, and does not capture specific behaviors that contribute to the risk of behaviors and outcomes. There are subpopulations that this methodology does not fully capture, including certain immigrant groups and some English language learners. In 2015, community organizations estimated undocumented immigrants to number 10,000 in New Bedford [29]. Information from these subpopulations will not be represented in administrative databases and may have distinct behavioral patterns, as newly arrived immigrants often reflect the health behaviors and outcomes of their home country, also known as the “healthy immigrant effect” [30]. Because BRFSS is a phone survey conducted in English, barriers to participation are access to a phone and English language proficiency. Children are also not captured because there is a lack of information on those under 18 years of age in administrative datasets despite the high interest and need for such databases.

Another limitation derives from constructing regression models using populations outside of New Bedford. While using the models created from the New Bedford data only would be a more accurate representation of New Bedford given potential differences in demographic patterns in other communities in Bristol County, the smaller sample size would contribute to greater statistical uncertainty. We tested our models with both datasets and found very similar results (Table S2). Many communities interested in this method may have much smaller populations, and the insights our models provided for community-level decision-making were robust to this choice, but this issue should be considered carefully when developing models in other locations, as demographic predictors may or may not generalize.

Our spatial microsimulation methods provide key data to inform decisions, but even setting aside the limited set of available predictors, they do not address the complexity of community issues that contribute to exercise, fruit or vegetable consumption, and diabetes rates. For example, identifying census tracts with low fruit and vegetable consumption to inform community action (such as adding a farmers market) may not lead to increased fruit and vegetable consumption. Improvement of diets is multi-faceted and complex—the produce being sold needs to be culturally relevant, affordable, and accessible [31]. Lastly, a limitation of the maps as a community information tool is that the maps may be misinterpreted by residents because the patterns in outcomes are related to sociodemographic risk factor patterns and not necessarily to the geography of where they live.

Nonetheless, our modeling approach adds value relative to the literature by leveraging spatial microsimulation and regression modeling methods to assist communities with decision-making using publicly available data, which tends to be more basic rather than nuanced. Therefore, our locally-informed models enable researchers to generate data from public databases for communities in a cost-effective, less resource intensive, and realistic manner, which could be followed up with more extensive surveys or local data collection if needed.

## 5. Conclusions

We conducted a community partner engaged analysis in the city of New Bedford, identifying health outcomes and behaviors of interest, developing spatial microsimulation models to characterize demographic and geographic patterns of those outcomes and behaviors, and sharing the results to inform community partner decision-making and resource allocation or attainment. The combination of complex simulation modeling, strong community partner engagement, and use of public administrative databases is a significant strength of this study and one that can generalize to other settings and situations where public health priorities are being determined. While the university and community partners in this study had a working relationship that predated this analysis, the relationships and mutual understanding matured through the collaborative process. The community partners broadened their scope of work and field of vision of community health and environmental planning, while the university partners’ understanding and appreciation of the needs and strengths of the community were deepened, which will allow future research in the city to be tailored to meet those needs. Our model platform can be expanded to address other variables of interest within New Bedford or to include other cities with interest in evaluating geographic and demographic patterns of key behaviors and health outcomes.

## Figures and Tables

**Table 1:** Multivariable regression coefficients for exercise, fruit and vegetable consumption, body mass index (BMI), and diabetes constructed from Behavioral Risk Factor Surveillance System (BRFSS) data for Bristol County, Massachusetts (2005–2010). BMI model is a linear regression, and other models are logistic regressions. Full models with standard errors are in Table S2.

Main Effect Coefficients for Each Modeled Outcome				
Covariate	Exercise ^a^	Fruit/vegetable ^b^	BMI	Diabetes ^c^
Sex				
Male	0.086 *	−0.29 *	0.036 *	0.21 *
Age				
18–29	0.40 *	−0.14	0.047 *	−1.65 *
30–39	0.18 *	−0.35 *	0.093 *	−0.93 *
40–49	0.072	−0.13	0.094 *	−0.29 *
50–59	−0.040	0.052	0.11 *	0.44 *
60–69	−0.077	0.062	0.11 *	0.72 *
70–79	−0.12 *	0.16	0.079 *	0.99 *
Race/ethnicity				
Black, non-Hispanic	−0.021	0.21	NS	0.071
Hispanic	−0.35 *	0.20	NS	0.19
Other (includes Asian)	0.24 *	−0.19	NS	−0.11
Income				
<$25,000	−0.20 *	0.051	0.02 *	0.27 *
$25,000–34,999	−0.014	−0.17 *	0.0098	−0.068
Education				
<High school	−0.26 *	−0.18 *	0.034 *	NS
High school	−0.077 *	0.000039	0.022 *	NS
Smoking				
Current	−0.30 *	−0.30 *	−0.05 *	−0.057 *
Former	0.096 *	0.078	0.0076	0.17 *
Alcohol				
At least one drink in past 30 days	0.25 *	NS	−0.030 *	−0.36 *
Exercise				
Any exercise in past 30 days	N/A	0.33 *	−0.043 *	−0.083 *
Fruit/vegetable consumption				
Five or more servings daily	N/A	N/A	−0.019 *	NS
BMI category				
Obese (BMI ≥ 30)	N/A	N/A	N/A	0.91 *
Overweight (30 > BMI ≥ 25)	N/A	N/A	N/A	0.054
Normal Weight (25 > BMI ≥ 18.5)	N/A	N/A	N/A	−0.53 *

^a^ Exercise: self-reported any exercise in the past 30 days, ^b^ Fruit/vegetable: self-reported 5 or more servings of fruit and vegetables daily, ^c^ Diabetes: self-reported ever being told they have diabetes by a doctor; Reference groups: female, age 80–99, White Non-Hispanic, income $35,000 and over, above high school education, never smoked, no alcohol in past 30 days, no exercise in past 30 days, <5 servings of fruits/vegetables daily, underweight (BMI < 18.5); * significant at *p* < 0.05; NS: not a significant predictor, *p*-value ≥ 0.05, predictor dropped from the final model; N/A: not considered in model (due to the order in which our regression models were built).

**Table 2:** Comparison of mean prevalence of exercising, fruit and vegetable consumption, and diabetes estimated from synthetic microdata compared to New Bedford and Massachusetts 2005–2010 BRFSS estimates for adults 21 years and older.

Outcome	New Bedford Synthetic Microdata Prevalence (95% CI)	New Bedford BRFSS Data, 2005–2010 Prevalence (95% CI) *	Massachusetts BRFSS Data, 2005–2010 Prevalence (95% CI) *
Exercise (self report in the past 30 days)	64.9 (62.5–67.2) %	66.3 (64.4–68.2) %	78.2 (77.8–78.6) %
Fruit and vegetable consumption (>5 daily)	17.9 (15.4–20.7) %	20.6 (18.2–22.9) %	27.5 (26.8–28.1) %
Diabetes (self reported doctor diagnosis)	11.1 (9.8–12.4) %	10.3 (9.3–11.4) %	7.4 (7.2–7.6) %

* Source: MA DPH, 2016. Note on MA DPH, 2016 data: estimates for New Bedford were calculated using statewide weights.

## Supplementary Materials

The following are available online at www.mdpi.com/1660-4601/14/7/730/s1, Table S1: Candidate predictors of exercise, fruit and vegetable consumption, BMI and diabetes from the literature compared to the predictors available in the BRFSS and New Bedford synthetic microdata, Table S2: Comparison of multivariable regressions coefficients predicting exercise, fruit and vegetable consumption, BMI and diabetes constructed from BRFSS data for Bristol County, MA (2005–2010) and New Bedford, MA (2005–2010); Bibliography for Literature Search.
