# Supplementary material for: Community-Engaged Modeling of Geographic and Demographic Patterns of Multiple Public Health Risk Factors
Source: Int J Environ Res Public Health. 2017 Jul 6;14(7):730. doi: 10.3390/ijerph14070730 (PMC5551168; doi:10.3390/ijerph14070730)
Supplement: Supplementary file 1 [file ijerph-14-00730-s001.pdf]

Table S1. Candidate predictors of exercise, fruit and vegetable consumption, BMI and diabetes from the literature compared to the predictors available in the BRFSS and synthetic microdata.

	Predictors	Selected Predictors Identified from Literature Review				Predictors Available in the BRFSS and Synthetic Microdata
		Exercise [1–15]	Fruit and vegetable consumption [16–22]	BMI [23–35]	Diabetes [36–45]	
Demographic and psychosocial	Sex	X	X	X	X	X
	Age	X	X	X	X	X
	Race/ethnicity	X	X	X	X	X
	Income	X	X	X	X	X
	Education	X	X	X	X	X
	Employment	X	X	X		X
	Smoking	X	X	X	X	X
	Alcohol	X		X	X	X
	Exercise		X	X	X	X
	Diet			X	X	X
	BMI	X	X		X	X
Social support		X	X			
Self-efficacy		X	X			
Marital status		X	X			
Motivation		X	X			
Family history diabetes					X	
Sleep		X		X		
Mental health		X		X		
Hypertension					X	
Hypocholesteremia					X	
Food Access			X	X		
Screen time (computer or TV)		X		X	X	
Green space		X		X		
Breastfeeding			X	X		

Table S2. Comparison of multivariable regressions coefficients predicting exercise, fruit and vegetable consumption, BMI and diabetes constructed from BRFSS data for Bristol County, MA (2005-2010) and New Bedford, MA (2005-2010).

Table S2. (a) Multivariable logistic regression for any exercise in past 30 days.

	Bristol County Model			New Bedford Model		
Covariate	Beta	Standard Error	p-value	Beta	Standard Error	p-value
Intercept	0.60	0.047	<.0001	0.62	0.067	<.0001
Sex						
Male	0.086	0.021	<.0001	0.11	0.040	0.0062
Age						
18-29	0.40	0.070	<.0001	0.34	0.12	0.0035
30-39	0.18	0.053	0.0005	0.29	0.096	0.0026
40-49	0.072	0.047	0.12	0.083	0.086	0.34
50-59	-0.040	0.043	0.35	-0.035	0.079	0.66
60-69	-0.077	0.045	0.087	-0.032	0.086	0.71

70-79	-0.12	0.053	0.020	-0.23	0.10	0.024
		Race/ethnicity				
Black, non-Hispanic	-0.021	0.096	0.83	0.051	0.12	0.68
Hispanic	-0.35	0.072	<.0001	-0.40	0.10	<.0001
Other (includes Asian)	0.24	0.083	0.0033	0.26	0.12	0.023
		Income				
< $25,000	-0.20	0.032	<.0001	-0.23	0.060	<.0001
$25,000-34,999	-0.014	0.040	0.72	-0.0035	0.073	0.96
		Education				
< High school	-0.26	0.036	<.0001	-0.24	0.062	0.0001
High school	-0.077	0.030	0.0075	-0.041	0.053	0.44
		Smoking				
Current	-0.30	0.032	<.0001	-0.27	0.058	<.0001
Former	0.096	0.030	0.0015	0.081	0.059	0.17
		Alcohol				
At least 1 drink in past 30 days	0.25	0.021	<.0001	0.22	0.040	<.0001

Bristol PROC LOGISTIC: exercise = sex + age + race + income + education + smoking + alcohol

New Bedford PROC LOGISTIC: exercise = sex + age + race + income + education + smoking +alcohol

Reference: female, age 80-99, White Non-Hispanic, 35k and over, above high school education, never smoked, no alcoholic drinks in past 30 days

Table S2. (b) Multivariable logistic regression of daily fruit and vegetable consumption:

	**Bristol County Model**			**New Bedford Model**		
Covariate	Beta	Standard Error	p-value	Beta	Standard Error	p-value
Intercept	-1.5	0.079	<.0001	-1.5	0.12	<.0001
		Sex				
Male	-0.29	0.033	<.0001	-0.16	0.065	0.016
		Age				
18-29	-0.14	0.10	0.17	-0.22	0.18	0.23
30-39	-0.35	0.083	<.0001	-0.18	0.15	0.24
40-49	-0.13	0.073	0.069	-0.049	0.14	0.72
50-59	0.052	0.065	0.43	0.040	0.13	0.75
60-69	0.062	0.071	0.38	-0.12	0.15	0.43
70-79	0.16	0.083	0.057	0.041	0.16	0.80
		Race/ethnicity				
Black, non-Hispanic	0.21	0.14	0.15	0.40	0.19	0.031
Hispanic	0.20	0.12	0.10	-0.04	0.18	0.84
Other (includes Asian)	-0.19	0.14	0.17	-0.26	0.20	0.20
		Income				
< $25,000	0.051	0.055	0.36	0.14	0.010	0.16
$25,000-34,999	-0.17	0.067	0.012	-0.11	0.13	0.37
		Education				
< High school	-0.18	0.067	0.0062	-0.11	0.11	0.32

High school	0.000039	0.050	0.99	-0.090	0.092	0.33
		Smoking				
Current	-0.30	0.058	<.0001	-0.28	0.11	0.0076
Former	0.078	0.049	0.11	0.031	0.096	0.75
		Exercise				
Any exercise in past 30 days	0.33	0.039	<.0001	0.28	0.071	<.0001

Bristol PROC LOGISTIC: fruit and vegetable consumption = sex + age + race + income + education + smoking + exercise

New Bedford PROC LOGISTIC: fruit and vegetable consumption= sex + smoking + exercise

Reference: female, age 80-99, White Non-Hispanic, 35k and over, above high school education, never smoked, no exercise in past 30 days

Table S2. (c) Multivariable Linear Regression Model of BMI:

	Bristol County Model			New Bedford Model		
Covariate	Beta	Standard Error	p-value	Beta	Standard Error	p-value
Intercept	7.8	0.011	<.0001	7.9	0.022	<.0001
		Sex				
Male	0.036	0.0048	<.0001	0.0095	0.0096	0.33
		Age				
18-29	0.047	0.012	<.0001	0.045	0.023	0.049
30-39	0.093	0.011	<.0001	0.092	0.022	<.0001
40-49	0.094	0.011	<.0001	0.086	0.021	<.0001
50-59	0.11	0.010	<.0001	0.12	0.020	<.0001
60-69	0.11	0.010	<.0001	0.10	0.021	<.0001
70-79	0.079	0.011	<.0001	0.065	0.022	0.0026
		Income				
< $25,000	0.021	0.0061	0.0005	0.017	0.011	0.13
$25,000-34,999	0.010	0.0076	0.20	0.0049	0.015	0.74
		Education				
< High school	0.034	0.0074	<.0001	0.023	0.013	0.095
High school	0.022	0.0055	<.0001	0.017	0.011	0.11
		Smoking				
Current	-0.051	0.0062	<.0001	-0.059	0.012	<.0001
Former	0.0076	0.0055	0.17	0.010	0.011	0.37
		Alcohol				
At least 1 drink in past 30 days	-0.030	0.0050	<.0001	-0.032	0.0098	0.0012
		Exercise				
Any exercise in past 30 days	-0.043	0.0053	<.0001	-0.048	0.010	<.0001
		Fruit and vegetable consumption				
5 or more servings of fruit daily	-0.020	0.0055	0.0007	-0.019	0.011	0.079

Bristol PROC GLM: Log(BMI) = sex + age + income + education + smoking + alcohol + exercise + fruit and vegetable consumption

New Bedford PROC GLM: Log(BMI)=age + education + employment + smoking + alcohol + exercise + fruit and vegetable consumption

Reference: female, age 80-99, 35k and over, above high school education, unemployed, never smoked, no alcoholic drinks in past 30 days, no exercise in past 30 days, 0-5 servings of fruits/vegetables dail

Table S2. (d) Multivariable logistic regression for diabetes.

	Bristol County Model			New Bedford Model		
Covariate	Beta	Standard Error	p-value	Beta	Standard Error	p-value
Intercept	-2.4	0.11	<.0001	-2.4	0.19	<.0001
		Sex				
Male	0.21	0.031	<.0001	0.14	0.057	0.017
		Age				
18-29	-1.65	0.22	<.0001	-1.4	0.30	<.0001
30-39	-0.93	0.12	<.0001	-0.99	0.20	<.0001
40-49	-0.29	0.087	0.0007	-0.25	0.15	0.08
50-59	0.44	0.068	<.0001	0.53	0.11	<.0001
60-69	0.72	0.067	<.0001	0.47	0.12	<.0001
70-79	0.99	0.074	<.0001	0.85	0.13	<.0001
		Race/ethnicity				
Black, non-Hispanic	0.071	0.14	0.62	0.17	0.18	0.36
Hispanic	0.19	0.11	0.074	0.22	0.15	0.14
Other (includes Asian)	-0.11	0.12	0.37	-0.30	0.17	0.09
		Income				
< $25,000	0.27	0.044	<.0001	0.30	0.081	0.0002
$25,000-34,999	-0.068	0.058	0.24	-0.11	0.11	0.33
		Smoking				
Current	-0.057	0.051	<.0001	-0.15	0.091	0.09
Former	0.17	0.042	<.0001	0.30	0.078	0.0001
		Alcohol				
At least 1 drink in past 30 days	-0.36	0.032	<.0001	-0.40	0.060	<.0001
		Exercise				
Any exercise in past 30 days	-0.083	0.031	0.0069	-0.0011	0.056	0.98
		BMI category				
Obese (BMI >=30)	0.91	0.090	<.0001	0.89	0.17	<.0001
Overweight (30>BMI >= 25)	0.054	0.091	0.55	-0.053	0.18	0.76
Normal Weight (18.5<= BMI <25)	-0.53	0.097	<.0001	-0.46	0.18	0.013

Bristol PROC LOGISTIC: diabetes = sex + age + race + income + smoking + alcohol + exercise + BMI category

New Bedford PROC LOGISTIC: diabetes= sex + age + income + smoking + BMI category

Reference: female, age 80-99, White Non-Hispanic, 35k and over, never smoked, no alcoholic drinks in past 30 days, no exercise in past 30 days, underweight (BMI <18.5)

Bibliography for Literature Search

1. Booth ML, Bauman A, Owen N, Gore CJ. Physical Activity Preferences, Preferred Sources of Assistance, and Perceived Barriers to Increased Activity among Physically Inactive Australians. *Prev Med (Baltim)*. 1997;26(1):131-137. doi:10.1006/pmed.1996.9982.
2. Boutelle KN, Jeffery RW, French SA, et al. Predictors of vigorous exercise adoption and maintenance over four years in a community sample. *Int J Behav Nutr Phys Act*. 2004;1(1):13. doi:10.1186/1479-5868-1-13.
3. Ferguson KJ, Yesalis CE, Pomrehn PR, Kirkpatrick MB. Attitudes, Knowledge, and Beliefs as Predictors of Exercise Intent and Behavior in Schoolchildren. *J Sch Health*. 1989;59(3):112-115. doi:10.1111/j.1746-

1561.1989.tb04675.x.
4. Giles-Corti B, Broomhall MH, Knuiman M, et al. Increasing walking: How important is distance to, attractiveness, and size of public open space? *Am J Prev Med*. 2005;28(2):169-176. doi:10.1016/j.amepre.2004.10.018.
5. Humpel N, Owen N, Leslie E. Environmental factors associated with adults' participation in physical activity: A review. *Am J Prev Med*. 2002;22(3):188-199. doi:10.1016/S0749-3797(01)00426-3.
6. Oka RK, King AC, Young DR. Sources of social support as predictors of exercise adherence in women and men ages 50 to 65 years. *Womens Health*. 1995;1(2):161-175. http://www.ncbi.nlm.nih.gov/pubmed/9373378. Accessed November 2, 2016.
7. Payne N, Jones F, Harris P. The impact of working life on health behavior: The effect of job strain on the cognitive predictors of exercise. *J Occup Health Psychol*. 2002;7(4):342-353. doi:10.1037/1076-8998.7.4.342.
8. Rodgers WM, Hall CR, Blanchard CM, McAuley E, Munroe KJ. Task and Scheduling Self-efficacy as Predictors of Exercise Behavior. *Psychol Health*. 2002;17(4):405-416. doi:10.1080/0887044022000004902.
9. Sallis JF, Zakarian JM, Hovell MF, Hofstetter CR. Ethnic, socioeconomic, and sex differences in physical activity among adolescents. *J Clin Epidemiol*. 1996;49(2):125-134. doi:10.1016/0895-4356(95)00514-5.
10. Sugiyama T, Leslie E, Giles-Corti B, Owen N. Associations of neighbourhood greenness with physical and mental health: do walking, social coherence and local social interaction explain the relationships? *J Epidemiol Community Health*. 2008;62(5):e9. doi:10.1136/JECH.2007.064287.
11. Sullum J, Clark MM, King TK. Predictors of Exercise Relapse in a College Population. *J Am Coll Heal*. 2000;48(4):175-180. doi:10.1080/07448480009595693.
12. Tappe MK, Duda JL, Menges-Ehrnwald P. Personal investment predictors of adolescent motivational orientation toward exercise. *Can J Sport Sci*. 1990;15(3):185-192. http://www.ncbi.nlm.nih.gov/pubmed/2257532. Accessed November 2, 2016.
13. Titze S, Stronegger W, Owen N. Prospective study of individual, social, and environmental predictors of physical activity: women's leisure running. *Psychol Sport Exerc*. 2005;6(3):363-376. doi:10.1016/j.psychsport.2004.06.001.
14. Tucker P, Gilliland J. The effect of season and weather on physical activity: A systematic review. *Public Health*. 2007;121(12):909-922. doi:10.1016/j.puhe.2007.04.009.
15. Tucker P, Irwin JD, Gilliland J, He M, Larsen K, Hess P. Environmental influences on physical activity levels in youth. *Health Place*. 2009;15(1):357-363. doi:10.1016/j.healthplace.2008.07.001.
16. Brug J, de Vet E, de Nooijer J, Verplanken B. Predicting Fruit Consumption: Cognitions, Intention, and Habits. *J Nutr Educ Behav*. 2006;38(2):73-81. doi:10.1016/j.jneb.2005.11.027.
17. GLANZ K, BASIL M, MAIBACH E, GOLDBERG J, SNYDER D. Why Americans Eat What They Do: Taste, Nutrition, Cost, Convenience, and Weight Control Concerns as Influences on Food Consumption. *J Am Diet Assoc*. 1998;98(10):1118-1126. doi:10.1016/S0002-8223(98)00260-0.
18. HAVAS S, TREIMAN K, LANGENBERG P, et al. Factors Associated with Fruit and Vegetable Consumption among Women Participating in WIC. *J Am Diet Assoc*. 1998;98(10):1141-1148. doi:10.1016/S0002-8223(98)00264-8.
19. Lin B-H, Morrison RM. Higher Fruit Consumption Linked With Lower Body Mass Index. *Food Rev*. 2002;25(3):28-32.
20. Nollen N, Befort C, Pulvers K, et al. Demographic and psychosocial factors associated with increased fruit and vegetable consumption among smokers in public housing enrolled in a randomized trial. *Heal Psychol*. 2008;27(3, Suppl):S252-S259. doi:10.1037/0278-6133.27.3(Suppl.).S252.
21. Rasmussen M, Krølner R, Klepp K-I, et al. Determinants of fruit and vegetable consumption among children and adolescents: a review of the literature. Part I: quantitative studies. *Int J Behav Nutr Phys Act*. 2006;3(1):22. doi:10.1186/1479-5868-3-22.
22. TRUDEAU E, KRISTAL AR, LI S, PATTERSON RE. Demographic and Psychosocial Predictors of Fruit and Vegetable Intakes Differ: Implications for Dietary Interventions. *J Am Diet Assoc*. 1998;98(12):1412-1417. doi:10.1016/S0002-8223(98)00319-8.
23. Albrecht SS, Gordon-Larsen P, Wang Y, et al. Ethnic Differences in Body Mass Index Trajectories from Adolescence to Adulthood: A Focus on Hispanic and Asian Subgroups in the United States. Votruba SB, ed. *PLoS One*. 2013;8(9):e72983. doi:10.1371/journal.pone.0072983.
24. Bruce MA, Sims M, Miller S, Elliott V, Ladipo M. One size fits all? Race, gender and body mass index among U.S. adults. *J Natl Med Assoc*. 2007;99(10):1152-1158. http://www.ncbi.nlm.nih.gov/pubmed/17987919. Accessed November 2, 2016.

25. Burke GL, Bild DE, Hilner JE, Folsom AR, Wagenknecht LE, Sidney S. Differences in weight gain in relation to race, gender, age and education in young adults: The CARDIA study. *Ethn Health*. 1996;1(4):327-335. doi:10.1080/13557858.1996.9961802.
26. Chou S-Y, Grossman M, Saffer H. An economic analysis of adult obesity: results from the Behavioral Risk Factor Surveillance System. *J Health Econ*. 2004;23(3):565-587. doi:10.1016/j.jhealeco.2003.10.003.
27. Gangwisch JE, Malaspina D, Boden-Albala B, Heymsfield SB. Inadequate Sleep as a Risk Factor for Obesity: Analyses of the NHANES I. *Sleep*. 2005;28(10):1289-1296.
28. Ghimire R, Green GT, Ferreira S, Poudyal NC, Cordell HK. Green Space and Adult Obesity Prevalence in the United States.
29. Goodman E, Whitaker RC. A Prospective Study of the Role of Depression in the Development and Persistence of Adolescent Obesity. *Pediatrics*. 2002;110(3).
30. Jackson AS, Stanforth PR, Gagnon J, et al. The effect of sex, age and race on estimating percentage body fat from body mass index: The Heritage Family Study. *Int J Obes Relat Metab Disord*. 2002;26(6):789-796. doi:10.1038/sj.ijo.0802006.
31. Jolliffe D. Overweight and poor? On the relationship between income and the body mass index. *Econ Hum Biol*. 2011;9(4):342-355. doi:10.1016/j.ehb.2011.07.004.
32. Luppino FS, de Wit LM, Bouvy PF, et al. Overweight, Obesity, and Depression. *Arch Gen Psychiatry*. 2010;67(3):220. doi:10.1001/archgenpsychiatry.2010.2.
33. Morland KB, Evenson KR. Obesity prevalence and the local food environment. *Health Place*. 2009;15(2):491-495. doi:10.1016/j.healthplace.2008.09.004.
34. Traversy G, Chaput J-P. Alcohol Consumption and Obesity: An Update. *Curr Obes Rep*. 2015;4(1):122-130. doi:10.1007/s13679-014-0129-4.
35. Vandelanotte C, Sugiyama T, Gardiner P, Owen N. Associations of leisure-time internet and computer use with overweight and obesity, physical activity and sedentary behaviors: cross-sectional study. *J Med Internet Res*. 2009;11(3):e28. doi:10.2196/jmir.1084.
36. Annis AM, Caulder MS, Cook ML, Duquette D. Family history, diabetes, and other demographic and risk factors among participants of the National Health and Nutrition Examination Survey 1999-2002. *Prev Chronic Dis*. 2005;2(2):A19. http://www.ncbi.nlm.nih.gov/pubmed/15888230. Accessed November 2, 2016.
37. Chan JM, Rimm EB, Colditz GA, Stampfer MJ, Willett WC. Obesity, Fat Distribution, and Weight Gain as Risk Factors for Clinical Diabetes in Men. *Diabetes Care*. 1994;17(9).
38. Colditz GA, Willett WC, Rotnitzky A, Manson JE. Weight Gain as a Risk Factor for Clinical Diabetes Mellitus in Women. *Ann Intern Med*. 1995;122(7):481. doi:10.7326/0003-4819-122-7-199504010-00001.
39. Herman WH, Ma Y, Uwaifo G, et al. Differences in A1C by Race and Ethnicity Among Patients With Impaired Glucose Tolerance in the Diabetes Prevention Program. *Diabetes Care*. 2007;30(10).
40. Hu FB. Globalization of Diabetes. *Diabetes Care*. 2011;34(6).
41. Hu FB, Leitzmann MF, Stampfer MJ, et al. Physical Activity and Television Watching in Relation to Risk for Type 2 Diabetes Mellitus in Men. *Arch Intern Med*. 2001;161(12):1542. doi:10.1001/archinte.161.12.1542.
42. Hu FB, Manson JE, Stampfer MJ, et al. Diet, Lifestyle, and the Risk of Type 2 Diabetes Mellitus in Women. *N Engl J Med*. 2001;345(11):790-797. doi:10.1056/NEJMoa010492.
43. Maty SC, Everson-Rose SA, Haan MN, Raghunathan TE, Kaplan GA. Education, income, occupation, and the 34-year incidence (1965-99) of Type 2 diabetes in the Alameda County Study. *Int J Epidemiol*. 2005;34(6):1274-1281. doi:10.1093/ije/dyi167.
44. Selby J V, Peng T, Karter AJ, et al. High rates of co-occurrence of hypertension, elevated low-density lipoprotein cholesterol, and diabetes mellitus in a large managed care population. *Am J Manag Care*. 2004;10(2 Pt 2):163-170. http://www.ncbi.nlm.nih.gov/pubmed/15005509. Accessed November 2, 2016.
45. Writing Group for the SEARCH for Diabetes in Youth Study Group D, Dabelea D, Bell RA, et al. Incidence of diabetes in youth in the United States. *JAMA*. 2007;297(24):2716-2724. doi:10.1001/jama.297.24.2716.
